# Atomic scale chemical tomography of human bone

**DOI:** 10.1038/srep39958

**Published:** 2017-01-05

**Authors:** Brian Langelier, Xiaoyue Wang, Kathryn Grandfield

**Affiliations:** 1 Department of Materials Science and Engineering, McMaster University, Hamilton, ON, L8S 4L7, Canada; 2 School of Biomedical Engineering, McMaster University, Hamilton, ON, L8S 4L7, Canada

## Abstract

Human bone is a complex hierarchical material. Understanding bone structure and its corresponding composition at the nanometer scale is critical for elucidating mechanisms of biomineralization under healthy and pathological states. However, the three-dimensional structure and chemical nature of bone remains largely unexplored at the nanometer scale due to the challenges associated with characterizing both the structural and chemical integrity of bone simultaneously. Here, we use correlative transmission electron microscopy and atom probe tomography for the first time, to our knowledge, to reveal structures in human bone at the atomic level. This approach provides an overlaying chemical map of the organic and inorganic constituents of bone on its structure. This first use of atom probe tomography on human bone reveals local gradients, trace element detection of Mg, and the co-localization of Na with the inorganic-organic interface of bone mineral and collagen fibrils, suggesting the important role of Na-rich organics in the structural connection between mineral and collagen. Our findings provide the first insights into the hierarchical organization and chemical heterogeneity in human bone in three-dimensions at its smallest length scale – the atomic level. We demonstrate that atom probe tomography shows potential for new insights in biomineralization research on bone.

The human skeletal system has a profound importance for our daily lives: acting as the main mechanism for coordinating mobility, and as the largest ion exchanger in our bodies to maintain homeostasis. Understanding the structure of human bone at all hierarchical levels has major implications for mineralization mechanisms, mechanical support, and assessment and treatment of bone pathologies. It is generally accepted that bone consists of two main components: Type I collagen, and carbonated hydroxyapatite crystals, with other minor constituents: water, non-collagenous proteins, such as proteoglycans, cells and blood vessels^1^,^2^. These main components self-assemble into mineralized collagen fibers that are used as the building blocks for higher-level architectures. The organization of these components and their subsequent arrangement into hierarchical levels has large implications for the structure-function relationships of bone, including its mechanical properties^3^,^4^. Many studies have focused on the relationship between the mineral and organic components of bone, highlighting the energetic basis for their interaction^5^, and mapping out their structure with various electron microscopies^6^,^7^. Yet the biomineralization mechanisms of human bone remain debated^8^, partly due to the inability to characterize both bone structure and composition at its building block level. The appeal of studies with high spatial and chemical resolution, such as that presented herein, is the possibility to elucidate mechanisms of mineralization, and the structure-function relationships that govern behavior of bone from the sub-nanometer scale up.

The characterization of the nanoscale structural and chemical architecture of bone embodies a number of challenges, particularly in three-dimensions. Established techniques, such as nano-computed tomography (nano-CT), can probe the 3D structure of bone on the order of 100 nm yet the constituents of bone, collagen fibrils and carbonated hydroxyapatite crystals, are orders of magnitude smaller^9^. Similarly, techniques such as NMR have the capability to probe chemical structures of bone to the molecular level, even in hydrated states. Recent work with this technique has shown strong evidence for the presence of citrate bridges between bone-mineral and collagen, however the technique lacks the site-specificity to accurately describe the exact locale of species detected^10^. Other techniques to probe chemical composition of bone on the molecular level, such as Raman and Fourier-Transform Infrared spectroscopy are widespread. These can be linked to identifying markers of bone crystallinity, aging and disease state, as well as to specific changes in composition, such as degree of carbonate substitution in bone mineral^11^. Advances towards probing 3D chemical composition and collagen orientation simultaneously have been made by employing polarized Raman spectroscopy^12^. However, it is important to note these analyses resolve molecular information only spatially at the micron scale, probing features such as osteonal and lamellar bone. Recently, focused ion beam (FIB) serial sectioning has surfaced as another potential 3D imaging modality for bone structure, however present studies^2^,^6^ report demineralized bone tissue and this modality is restricted to the resolution limits of scanning electron microscopy (SEM). Indeed the transmission electron microscope (TEM) provides a suitable approach to nanoscale imaging, with possibilities in both 2D and 3D with electron tomography. Yet the inherent energy and spatial resolution limits of analytical spectroscopic techniques, such as energy dispersive x-ray spectroscopy (EDX) and electron energy loss spectroscopy (EELS) on beam-sensitive bone, have prevented the possibility for combined TEM and chemical analysis in 3D at the nanometer level.

To circumvent these challenges, we demonstrate herein that correlative compositional contrast imaging in the TEM with atom probe tomography (APT) provides a both a structural and chemical nanotomography of human bone. This technique has enabled us to perform the first APT on human bone to identify chemical heterogeneities and localization of trace elements.

APT is based on the successive field evaporation of atoms as ions from the surface of a specimen within an electric field, which are then collected by a lateral (*x, y*) position-sensitive detector. Field evaporation is triggered in pulses, which allows for both the sequence of evaporation and ion time-of-flight to be recorded for each ion. From these parameters, the original *z*-coordinates of the ions and an atomic mass-to-charge (*m/z*) spectrum can be determined. The data produced by an APT experiment can therefore be reconstructed to provide a 3D model of the analyzed volume, detailing the spatial and chemical features of the sample with sub-0.3 nm spatial resolution to 1 ppm chemical sensitivity^13^.

Traditionally, field evaporation in atom probe experiments was triggered by a pulsed electric field, which limited the type of specimens suitable for this technique to materials with good electrical conductivity and reasonable ductility (i.e. metals). However, the development of laser-pulsing techniques has widened the applicability of APT to nearly all manner of solid material^13^,^14^. The detailed investigation of apatites and biominerals with APT was first pioneered by the group of Joester *et al*. They have reported APT on hydroxyapatite, elephant dentin, rat femur cortical bone, and dental enamel, for example^15^,^16^,^17^,^18^. Since their successful demonstration of APT as a powerful characterization technique for bone-like materials and biominerals, the application of APT to other bone-related research, such as the investigation of implant-bone interfaces, has been noted^19^,^20^.

Although APT has been demonstrated on several biominerals, human bone presents unique challenges for successful APT analysis, including its high organic content (~35 wt%) and therefore chemically and crystallographically heterogeneous structure, not to mention its complex ionic and covalent bonding and its low conductivity. The system used for the current analysis utilized a pulsed 355 nm UV laser to illuminate the bone specimen tip in a static DC electric field, and promote field evaporation of surface atoms. Unfortunately, as human bone is a relatively poor conductor of heat at room temperature (0.16–0.34 W/m°C^21^), it is relatively slow to cool down following heating by the laser pulses, even at the low cryogenic temperatures used in APT. This means that in many cases the timing of atomic evaporation may substantially lag behind the timing of the laser pulse. This effect degrades the mass resolving power of the technique and leads to so-called thermal tails trailing form the ion peak to higher mass-to-charge values in the spectra. An attempt was made to optimize the laser pulse energy to minimize thermal tails while keeping background counts low, which yielded values lower than in previous reports of similar organic materials^15^,^16^. However, the very heterogeneous and highly organic nature of bone has generally made it difficult to identify the most optimum operating parameters, as so clearly demonstrated for other less heterogeneous bone-like materials, like hydroxyapatite. To highlight the complexity of achieving APT data on human bone, in this work more than 10 samples of human bone were prepared and analyzed in the atom probe, of which only ~60% of samples yielded more than 1 × 10^6^ ions, and only ~25% yielding more than 3 × 10^6^ ions. This paper primarily reports data from 3 of these samples, representing approximately 13 × 10^6^ total collected ions. Therefore, while a sufficient amount of data for analysis was obtained, the sample yield remained too low to allow for clear optimization of many APT operating parameters.

## Results and Discussion

Human bone tissue from the maxilla was shaped into a sharpened nano-pillar for TEM and APT using an *in-situ* FIB technique (Fig. S1). Z-contrast images were recorded on a high-angle annular dark-field (HAADF) detector to provide compositional contrast for structural cues. Using the same sample, APT unveiled the chemical distributions at the near sub-nanometer level, which could then be correlated with the electron microscopic images. The combination of these techniques enables us to report bone architectures and chemical distributions with unparalleled spatial accuracy and confidence.

**Figure 1:**
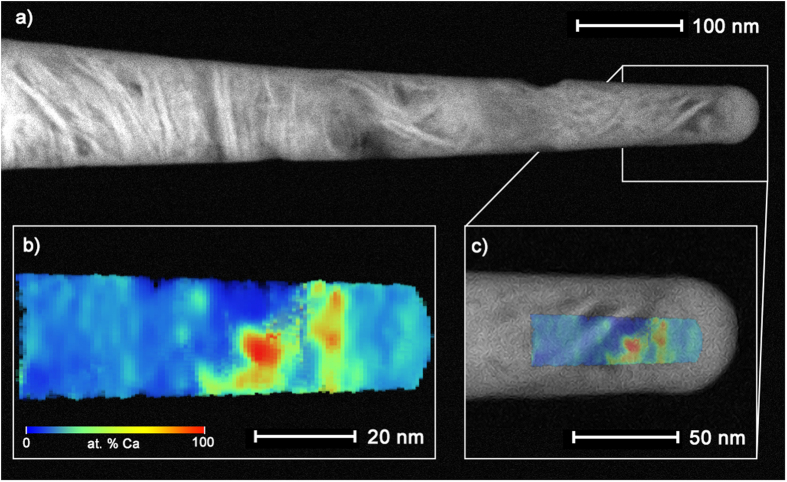
Correlative electron microscopy and APT reveal structural and chemical distributions in bone. Reconstruction parameters were optimized for detector and evaporation efficiency by utilizing the structural information from STEM HAADF imaging of the bone tip before APT (**a**), to produce the Ca concentration gradient map from APT analysis (**b**), shown overlapped in (**c**). Regions rich in Ca (red) correspond well to the plate like crystals imaged in STEM (bright needle-like features).

In this work, we push the analysis of human bone to the smallest length scale possible today-to the individual atoms that compose the tissue. Indeed, the complexity associated with APT experiments and data reconstruction with bone is compounded by its highly heterogeneous nature, as most standard APT theory and reconstruction algorithms are based on homogeneous materials. In this regard, the importance of correlative microscopies is highlighted. By comparing to STEM images of the specimen prior to APT analysis (e.g. a typical dataset shown in Fig. 1), we can measure the shank half angle accurately to assist with reconstruction, and do not have to depend on the relation between applied DC voltage and tip radius, which can be irregular for non-conductive materials. To improve the spatial accuracy of the reconstructions, the evaporation field would be estimated such that the Ca-rich features in the reconstruction were consistent with the bright contrast regions in the STEM images. The data acquisition and reconstruction procedures are further elaborated on within the Materials and Methods section of the manuscript. In addition, comparison with features in the STEM image enabled us to ascertain accurate reconstruction parameters to correctly distribute elements in 3D space. For example, the HAADF STEM image (Fig. 1a) can be easily interpreted based on its compositional contrast – the platelet-like bright rods are the calcium and phosphate-rich bone mineral crystals, seen in this image oriented slightly at an angle to the long axis of the APT needle. Less dense, organic-rich regions are darker in contrast. Knowledge of these features noted in the STEM image were key for optimizing the reconstruction parameters for the APT dataset overlaid in Fig. 1b and c. The reconstruction parameters generated from correlative data could also be applied to datasets when correlative data was not available.

**Figure 2:**
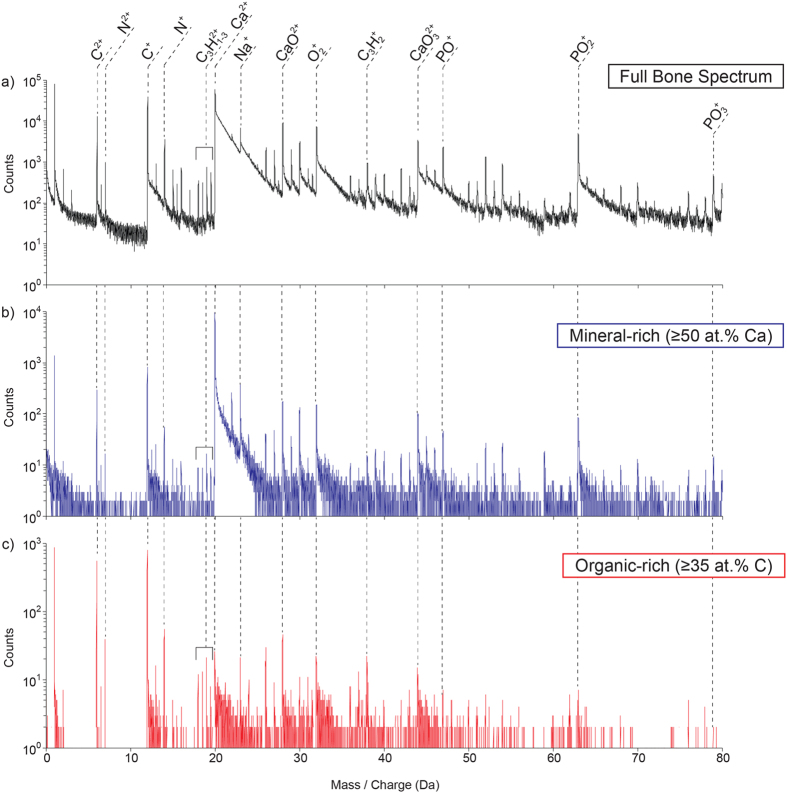
APT mass spectra of bone. Typical mass spectra for an APT analysis of human bone (**a**), as well as subvolumes corresponding to mineral Ca-rich (**b**), and matrix C-rich phases (**c**). The sub-volumes in (**b,c**) were selected as regions with ≥50 at.% Ca, and ≥35 at.% C, respectively. Select ionic species are also indicated on the spectra.

**Figure 3:**
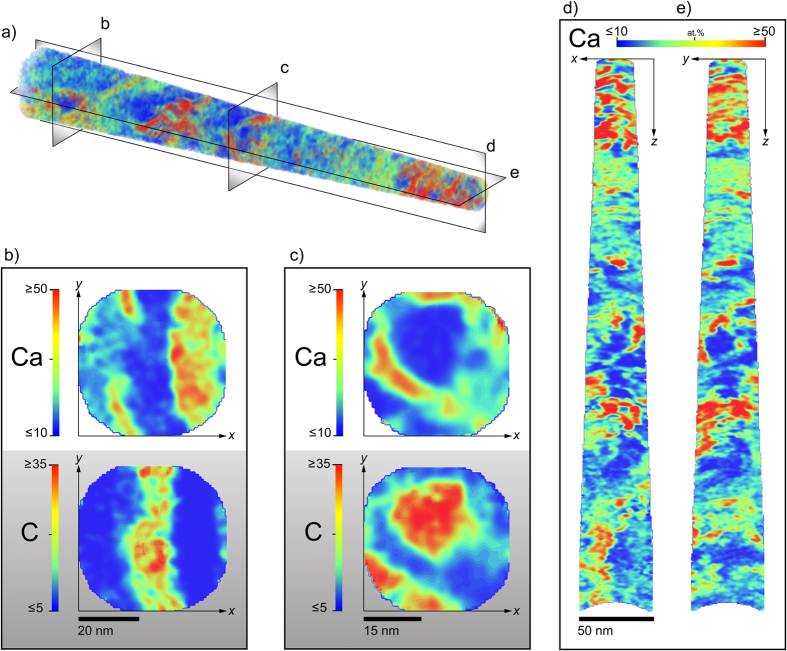
Mineral gradients in bone. 3D rendering shows the volume concentration of Ca, representing bone mineral, throughout a human bone specimen (**a**). Sections perpendicular to the specimen axis (**b,c**) and orthogonal sections parallel to the specimen axis (**d,e**) reveal details in structure variation. Sections perpendicular to the specimen axis highlight (**b**) alternating mineral and collagen-rich bands (shown by complementary C concentration maps), and (**c**) mineral rich regions surrounding circular organic-rich collagen fibrils, potentially locations of extrafibrillar mineral. Plate like crystals are clear in (**d,e**) showing alternating Ca-rich and deficient clusters that are on the order of 50–70 nm wide.

APT spectra from several bone specimens were collected; a representative spectrum highlighting the chemical complexity is shown in Fig. 2, for the full bone specimen (Fig. 2a) and sub-volumes related to mineral and matrix regions (Fig. 2b and c, respectively). Reconstruction of these spectra into gradient maps (e.g. Figs 1 and 3) provides a convenient way to visualize the compositional and spatial mineral-matrix relationships, which are less easy to visualize in 3D atom maps or isoconcentration surfaces due the dense heterogeneity of bone. Table 1 reports the overall composition of human bone as measured by APT and averaged over all datasets. Values are in close agreement with anticipated concentrations based on the assumption that the mineral phase in bone is hydroxyapatite. The Ca:P atomic ratio is measured to be 2.26 ± 0.51, near to the stoichiometric ratio of 1.67 in hydroxyapatite. Our datasets generally exhibit smaller thermal tails and lower background than many other bone-like minerals investigated, and thus show significantly more peaks above background. The unambiguous identification of many of these smaller peaks is very challenging, as there is typically an abundance of possible complex ions, and few supporting peaks of other isotopes that can be used confirm ranging. Additionally, it is still expected that many other peaks are present below the background or in the major thermal tails, and cannot be accounted for. This challenge with ranging ions is exemplified for P-related ions, where the low contribution to the Ca:P ratio is likely due to unranged P-containing compounds, either unidentified or lost to the background. Furthermore, some composition variation may be due to ambiguity in ranging for molecular ions whose peaks overlap due to equivalent mass:charge ratios, such as P_2_O_4_^2+^ which overlaps with PO_2_^+^. Unfortunately, standards for ranging these ions do not presently exist.

### Mineral-matrix organization

**Figure 4:**
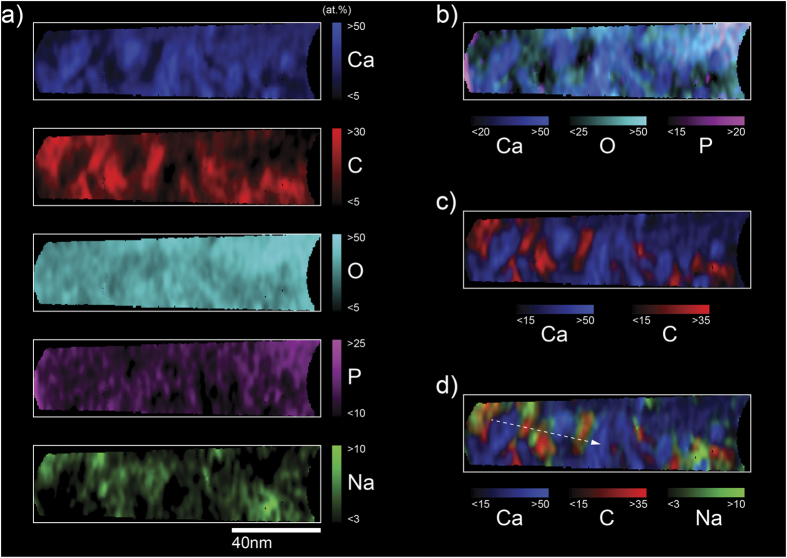
Composition mapping of major and minor elements in human bone by APT. The main components of bone (Ca, C, O, P and Na) from an 8 nm thick APT volume section (**a**). The mineral-rich elements of Ca, O, and P (**b**), are predominantly spatially inverse from regions of organic collagen fibrils (C-rich), as shown in (**c**). The Na concentration map overlaid onto the Ca, C concentration maps in (**d**) suggests the co-localization of Na with the organic components of bone, and at organic-inorganic interfaces. The dashed line in (**d**) indicates the region plotted in Fig. 5.

Our findings emphasize the plate-like structure of hydroxyapatite crystals, best highlighted in Ca-concentration gradient maps from a dataset of over 10 × 10^6^ ions, shown in Fig. 3. We find that the arrangement of Ca-rich mineral structures depends on their location or orientation within the sample. They appear as plates adjacent to C-rich regions (Fig. 3b) or as circular-like shapes encompassing C-rich and N-rich (not shown) regions that resemble fibrillar like collagen structures, although slightly smaller in diameter (Fig. 3c). This finding, and the general observation that the locations of Ca and C-rich regions are complementary to one another (Fig. 4), could further support the theory that some of the mineral component of bone exists as extrafibrillar mineral exterior to the gap zones in collagen fibril arrangement^22^,^23^, also described as mineral lamellae in other works^7^. Since the orientation of collagen in this bone sample from the maxilla is difficult to predetermine, we believe that future studies that restrict APT sample geometry to known collagen arrangements, such as in long bones, would aid in deciphering the location of hydroxyapatite within or exterior to collagen fibrils. To better visualize the overlay of organic matrix (C-rich) and inorganic mineral (Ca-rich) components, we refer readers to the Movie S1.

### Co-localization of elements

**Figure 5:**
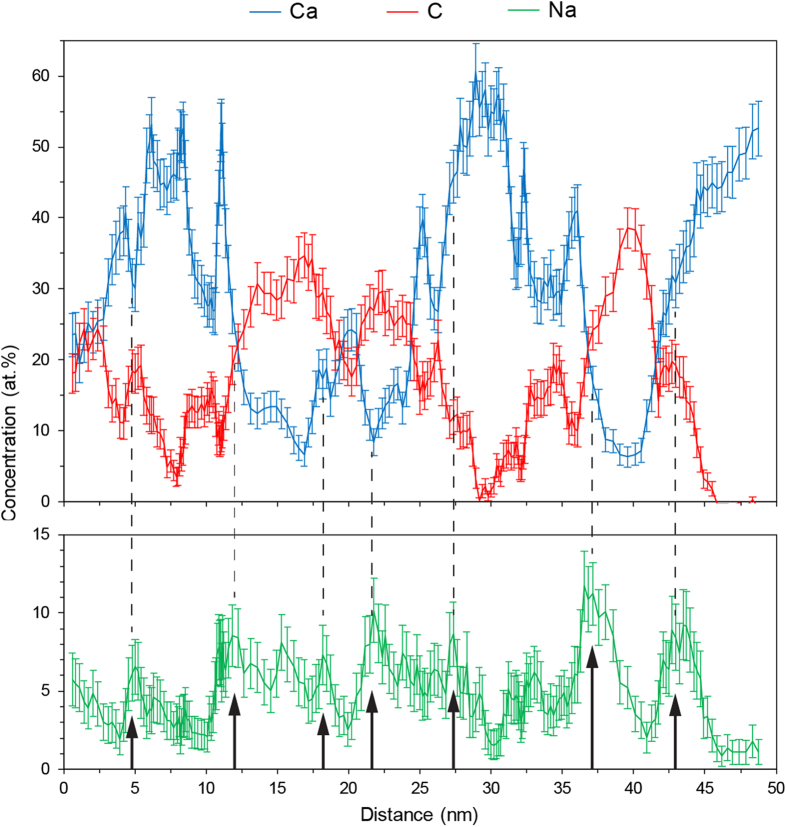
Composition profiles of Ca, C and Na. 1-D atomic concentration profiles for Ca, C and Na show that the concentrations of Ca and C are revealed to fluctuate opposite to each other, according to the relative amounts of mineral and organic phases being sampled. Na is predominantly co-localized with C, but frequently exhibits concentration peaks at mineral-organic interfaces (as indicated by arrows). These profiles were obtained by sampling the dataset shown in Fig. 4d, using a 5 × 5 × 50 nm sub-volume.

Bone contains many trace elements such as Na, Mg, F and Sr. Studies with secondary ion mass spectroscopy (SIMS) have suggested that Na is located within the organic phase of bone^24^. APT of other mineralized tissues, including elephant tusk dentin and the invertebrate chiton, have detected the co-localization of Na^+^ and Mg^2+^ ions with collagen, and attribute this to the presence of specific ion binding proteins or proteoglycans located in organic fibers^15^,^16^. Herein, Na has been found in throughout the bone, but is most concentrated in regions co-localized with the C-rich organic collagenous and non-collagenous protein components of bone (Fig. 4). When we plot compositions across a linear region of the reconstructed volume (Fig. 5), we can identify organic-inorganic boundaries as the regions showing sharp changes in composition between mineral (Ca-rich) and matrix (C-rich) regions, shown in the red and blue curves of Fig. 5. Adding Na to this plot (green curve, Fig. 5), we note that there are consistently slight enrichments in Na at the organic-inorganic interfaces, as marked by arrows (Fig. 5). While the localization of Na is clear in this work, similar conclusions for Mg are much more difficult to draw due to ambiguity in APT mass spectra caused by overlap between the major peaks of Mg and C in the mass spectra. Further explained figuratively in Fig. S2, the dominant peaks for Mg and C are ^24^Mg^2+^ and ^12^C^+^, which both appear at a mass:charge value of 12 Da. It could be confirmed that Mg and C co-localize by analyzing the location of ^24^Mg^+^ ions at 24 Da, but that peak may also exhibit overlap with C, in the form of ^12^C_2_^+^ ions. As there are 3 possible isotopes for Mg: ^24^Mg, ^25^Mg, and ^26^Mg, the minor isotopes of ^25^Mg and ^26^Mg could show peaks free of overlap, and confirm the co-localization of Mg with C. The ^25^Mg^2+^ peak at 12.5 Da is observed for the Ca-rich regions, confirming the presence of trace amounts of Mg in the mineral component of the bone. However, the opposite is found for the C-rich collagen, where that peak is not observed at a sufficient level to appear from behind the thermal tail of the major ^12^C^+^ peak at 12 Da. Therefore, in light of the uncertainty in identifying Mg in the collagen component, we are wary of drawing conclusions on the co-localization of Mg with C as seen in APT of other biominerals. Conversely, the peak for ^23^Na^+^ at 23 Da may also be somewhat overlapped, in this case by the ^40^Ca^2+^ thermal tail extending from 20 Da. However, we can confidently claim co-localization of Na and C because the ^23^Na^+^ is found to be enriched in areas of the collagen component, precisely where ^40^Ca^2+^ is not. Therefore, within these regions of low Ca concentration, the ^23^Na^+^ ion peak unambiguously represents Na, and there is no significant overlap with ^40^Ca^2+^. A full list of ranged ion species is found in Table S1. Our findings clearly demonstrate that APT is capable of detecting trace elements such as Mg and Na, and we can confidently state that Na is segregated into areas associated with organic-rich regions, and further enriched at organic-inorganic boundaries, while Mg can only be confirmed within the mineral phase, likely as Mg-substituted bone mineral.

While related results from other biomineral-based specimens are interesting, the significance of the current findings in human bone, compared to invertebrates and other vertebrates with drastically different skeletal structures than humans, is marked. APT is presently the only technique available to confirm the elemental heterogeneities we have shown in human bone at the near sub-nanometer length scale. It is important to note that in this study, human bone from the maxilla was not retrieved in a known orientation, i.e. not perpendicular or parallel to any loading or known anatomical feature. In the future, selecting a specimen from a very well characterized anatomical location could provide easier interpretation of APT data. For example, collagen fibrils and bone mineral generally align along the long-axis of long bones – providing this *a priori* information into the assignment of structures would simplify ambiguity in reconstruction.

## Conclusions

This work demonstrates the first successful APT of human bone, further improved by the correlation to STEM images to ensure accurate reconstruction. This near-atomic scale view highlights chemical heterogeneities and trace element detection of Mg and Na in human bone. The advanced resolution and three-dimensional chemical mapping capabilities of APT provide a platform for further investigation of other mineralized tissues, particularly with ideally prepared samples and operating conditions.

The low detection limits of APT open a new realm of characterization possibilities. In particular, it offers the opportunity to investigate the role of chemical heterogeneities within bone tissue at mineralization fronts, including both natural, i.e. osteonal structures, and engineered, i.e. implant-bone interfaces^19^,^25^. Furthermore, with continued technical advancements in APT, such as development of cryo-APT, we may be able remove ambiguity introduced by sample preparation, such as dehydration, of bone with the analysis of cryogenically frozen bone. This work has also highlighted the complex challenges still remaining with the successful acquisition and interpretation of APT data from such highly heterogeneous and organic-based non-conductive materials. Further development towards standards for data analysis of such materials is needed.

This successful APT of human bone, which highlighted the co-localization of Na with organic components and at inorganic-organic interfaces, and the detection of trace elements of Mg in bone mineral, sets the stage for further work in more complex scenarios of biomineralization. It is clear that a multitude of trace biomineralization details could be unveiled by further exploiting the chemical and spatial sensitivity of APT of human bone as we’ve demonstrated herein.

## Methods

### Bone sample preparation

The human bone used in this study was received with ethical approval from Biobank 513 at the Department of Biomaterials, University of Gothenburg, Sweden upon the removal of a fractured dental implant in place for 47 months in the maxilla of a 66-year old female patient (Courtesy of Drs. Thomsen and Palmquist) and studied with ethical approval from the Integrated Research Ethics Board at McMaster University, all experiments were performed in accordance with relevant guidelines and regulations. The sample was fixed in formalin, dehydrated in a graded series of ethanol and embedded in plastic resin (LR White, London Resin Company, UK) according to a previous publication^26^. The embedded bloc was cut longitudinally and a bone region was selected for further study. APT on the LR White resin only was conducted to assist in eliminating ambiguities in ranging the bone dataset, and is included in Fig. S3.

### STEM and APT sample preparation

Needle-shaped samples for correlative STEM imaging and APT were produced using a dual-beam focused ion beam (FIB) instrument (NVision 40, Carl Zeiss, Germany) and established protocols for APT sample production^27^, Fig. S1. A site in mature lamellar bone, away from large features such as blood vessels, cement lines and osteocytes, was selected by viewing the specimen with secondary and back-scattered electron imaging in the FIB-SEM. A layer of tungsten (10 μm × 2 μm × 0.5 μm) was deposited to protect the site of interest prior to rough milling of trenches and lift-out of a wedge-shaped sample. Using tungsten deposition, the lift-out wedge was either attached to the top of Si posts (CAMECA Scientific Instruments, Madison, WI) or to the top of electropolished tungsten wires mounted in 1.8 mm copper tubes. The mounted wedges were annularly milled into needles with diameters ranging between 56 nm and 180 nm. Initial milling was done using a 30 kV beam and successively lower currents (150-10 pA). A 10 kV 80 pA beam was used for final sharpening to minimize any potential damage or Ga ion implantation.

### STEM methods

The needle-shaped samples produced by FIB on tungsten wires were mounted in a Model 2050 on-axis rotation tomography holder (E.A. Fischione Instruments, Inc., Export, PA) and imaged in a Titan 80–300 TEM (FEI Company, The Netherlands) operated at 300 kV using a high-angle annular dark-field (HAADF) detector. To avoid sample damage prior to APT, only three images were recorded at −60°, 0°, and +60° tilt at a magnification of 160 k times.

### APT methods

Experiments on the needle-shaped specimens were conducted using a LEAP 4000XHR (CAMECA Scientific Instruments, Madison, WI). An ultraviolet laser pulse (λ = 355 nm) was used to incite field evaporation from the sample. Samples would frequently fracture before yielding a reasonable amount of data, with ~60% of samples yielding more than 1 × 10^6^ ions, and only ~25% yielding more than 3 × 10^6^ ions. The low yield of samples made optimization of experimental parameters difficult, so sample temperature (~43 K) and laser pulse rate (160 kHz) were kept constant, but the evaluation of laser pulse energy was attempted. Laser energies of 16, 75, 90, and 120 pJ were used for analysis, with 75 pJ and 90 pJ appearing to both yield the best quality data, considering both background counts (Fig. S4) and the “thermal tails” created by slow cooling of the specimen following a laser pulse (Fig. S5). Compositions appear consistent within this range of laser energies (Fig. S6); however, the small number of analyzed datasets makes it difficult to separate trends in the data from natural variability between samples. The pressure of the analysis chamber was <3.7 × 10^−9^ Pa. A target evaporation rate of 0.005 ions/pulse was maintained through varying the electric field on the sample, by means of controlling the DC voltage applied to the sample (typically 1.5–3.5 kV). Data was reconstructed using the Integrated Visualization and Analysis Software package (IVAS v3.6.6, CAMECA Scientific Instruments, Madison, WI). The reconstruction assumes the shape of a hemispherical tip on a truncated cone, with the shank half angle measured from STEM images. To improve the spatial accuracy of the reconstructions, the evaporation field would be estimated such that the Ca-rich features in the reconstruction were consistent with the bright contrast regions in the STEM images. The average evaporation field for human bone was determined to be *F* = 15 V/nm. The initial radius was calculated using the initial DC voltage applied to the specimen, given this evaporation field. A field factor of *k* = 3.3 and an image compression factor of ζ = 1.65 were used for all reconstructions. The average atomic volume was calculated from the hydroxyapatite unit cell (excluding H atoms)^15^. A high percentage of detected ions (70–80%) appear in the mass spectrum not as part of main ion peaks, but in the thermal tails following major peaks. To avoid ambiguity in assigning ionic species, these thermal tails were left un-ranged, and only the main ion peaks were used for reconstruction. This large discrepancy between detected and reconstructed ions was accounted for by modifying the detection efficiency parameter during the reconstruction. For most datasets, a value of η = 0.09 was used. Unidentified peaks from the mass spectrum were included in the reconstruction, which accounted for <8% of the total ranged ions. Where possible, unidentified ions are indicated as being found coincident with either the mineral (i.e. Ca-rich) or organic (i.e. C-rich) regions of the datasets, Table S1. For the analysis of elemental distributions, all identified ion peaks were used, with complex ions decomposed into their atomic components. See Table S1 for a complete list of ranged ions.

## Additional Information

**How to cite this article:** Langelier, B. *et al*. Atomic scale chemical tomography of human bone. *Sci. Rep.* **7**, 39958; doi: 10.1038/srep39958 (2017).

**Publisher's note:** Springer Nature remains neutral with regard to jurisdictional claims in published maps and institutional affiliations.

## Supplementary Material

Supplementary Video 1

Supplementary Information

## Figures and Tables

**Table 1:** Composition of human bone as measured by APT.

Element	Concentration from APT (at.%)
O	38.81 ± 4.18
Ca	27.44 ± 3.30
C	17.97 ± 6.19
P	12.28 ± 1.02
N	2.86 ± 0.35
Na	2.52 ± 0.80
Mg	0.15 ± 0.04

Average concentrations of elements detected in human bone from APT data, with results weighted based on the total number of ions in each acquired dataset.
