# Supplementary material for: Atomic scale chemical tomography of human bone
Source: Sci Rep. 2017 Jan 5;7:39958. doi: 10.1038/srep39958 (PMC5215514; doi:10.1038/srep39958)
Supplement: Supplementary Information [file srep39958-s2.pdf]

Atomic scale chemical tomography of human bone

*Brian Langelier[1], Xiaoyue Wang[1], Kathryn Grandfield[1]†**

[1]Department of Materials Science and Engineering, McMaster University, Hamilton, ON, L8S 4L7, Canada; and †School of Biomedical Engineering, McMaster University, Hamilton, ON, L8S 4L7, Canada

SUPPLEMENTARY MATERIALS:

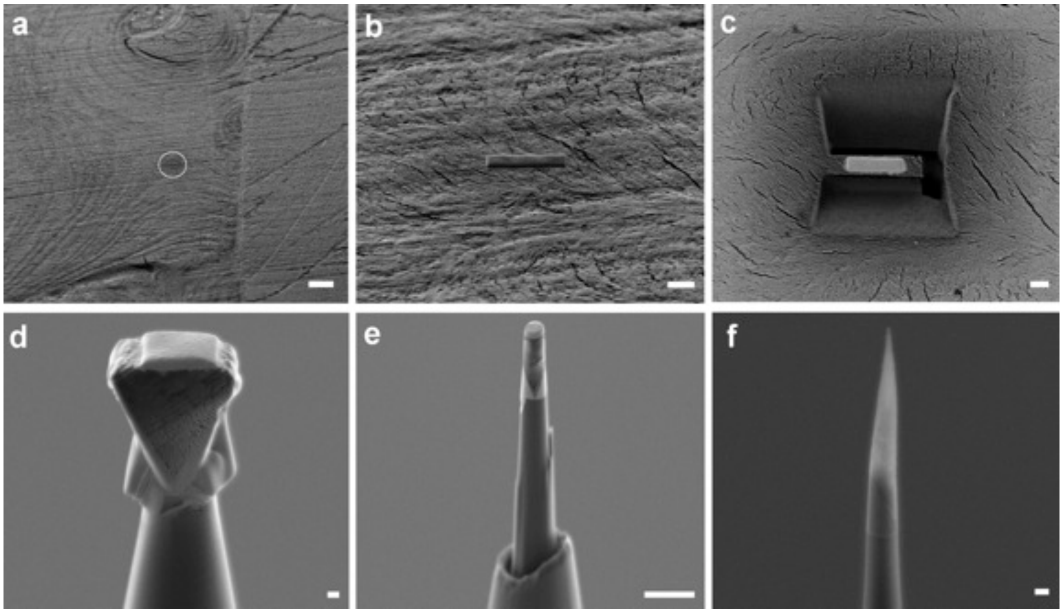

Figure S1. Focused ion beam sample preparation method. Following (a-f): site selection, protection with tungsten deposition, milling of rough trenches, lift-out and attachment of wedge to the tip of an electropolished W needle, and annular milling to a final needle-shaped pillar for APT. Scale bars (a) 40 μm, (b-e) 4 μm, (f) 200 nm.

Table S1. Ranged ion assignments from APT mass spectra

Mass/Charge (Da)	Ion
1	H^+
2	H_2^+
3	H_3^+
6, 6.5	C^{2+}
7	N^{2+}
12, 13	C^+
12.5	Mg^{2+}
14	N^+
15	NH^+
15.5	P^{2+}
16	O^+
17	OH^+
18	H_2O^+
18.5	C_3H^{2+}
19	$C_3H_2^{2+}$
19.5	$C_3H_3^{2+}$
20, 22	Ca^{2+}
23	Na^+
24	Mg^+
26	P_2O^{3+}
27	NaP^{2+}
27.5	$NaPH^{2+}$
28	CO^+
29	$CaPO^{3+}$
30	NO^+
31	P^+
31.5	P_2H^{2+}
32	$O2^+$
36	CaO_2^{2+}
37	C_3H^+
38	$C_3H_2^+$
39	P_2O^{2+}
40	Ca^+
41	*Unidentified*[1]
42	$Ca_3O_3^{4+}$
43	CP^+
43.5	$CaPO^{2+}$
44	CaO_3^{2+}
45	*Unidentified*[1]
46	P_2NO^{2+}
47	PO^+
48	Ca_2O^{2+}
49	*Unidentified*
50	*Unidentified*
50.5	*Unidentified*
51	*Unidentified*
52	CaO_4^{2+}
53	$Ca_2PO_3^{3+}$
54	*Unidentified*[1]
55	P_2Ca^{2+}
56	CaO^+
57	$CaOH^+$
58	*Unidentified*
59	*Unidentified*
60	*Unidentified*
61	*Unidentified*[2]
62	P_2^+
63	PO_2^+
64	PO_2H^+
66	*Unidentified*[1]
67	*Unidentified*
68	*Unidentified*[1]
69, 71	Ga^+
70	*Unidentified*[1]
72	CaO_2^+
73	*Unidentified*
74	*Unidentified*
75	*Unidentified*
76	*Unidentified*[2]
78	P_2O^+
79	PO_3^+
80	*Unidentified*[1]
81	*Unidentified*[1]
82	*Unidentified*
83	*Unidentified*
88	*Unidentified*
89	*Unidentified*
90	*Unidentified*
91	*Unidentified*[1]
92	*Unidentified*
94	*Unidentified*
96	*Unidentified*
100	*Unidentified*[2]
102	*Unidentified*
102.5	*Unidentified*[1]
103	*Unidentified*
105	*Unidentified*[1]
106	*Unidentified*[1]
107	*Unidentified*[1]
119.5	$CaPO_3^+$
134	$P_4O_9^{2+}$
142	$P_2O_5^+$
143	$P_2O_5H^+$
158	*Unidentified*[1]
205	*Unidentified*

[1]*Unidentified ion most common in mineral regions*

[2]*Unidentified ion most common in organic regions*

Note: Unidentified ions correspond to < 8 ionic % of total ranged ions.

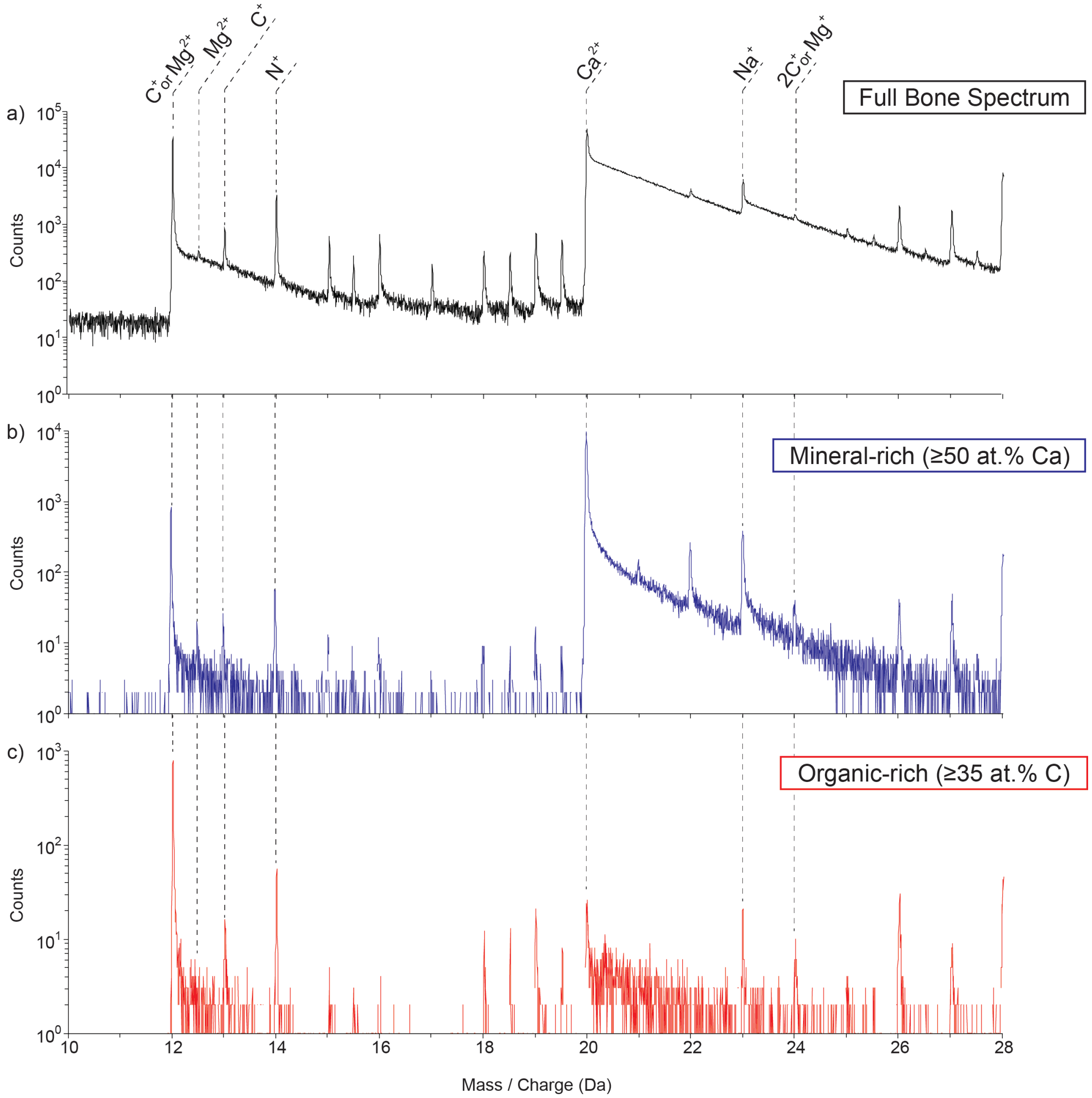

Figure S2. APT mass spectra over 10-28 Da range typical for human bone (a), as well as sub-volumes corresponding to Ca-rich mineral (≥ 50 at. % Ca) (b), and C-rich organic matrix (≥ 35 at. % C) phases (c). Select ionic species are also indicated on the spectra. The presence of the $^{23}Na^{+}$ peak at 23 Da shows that Na is detected in both the mineral and organic phases, but appears at relatively higher levels in the latter. Trace Mg is detected in the mineral, but cannot be disambiguated from C in the organic matrix phase due to overlap between $^{24}Mg^{2+}$ and $^{12}C^{+}$ at 12 Da, and $^{24}Mg^{+}$ and $^{12}C_2^{+}$ at 24 Da, and the $^{25}Mg^{2+}$ peak at 12.5 Da not appearing above the thermal tail for the $^{12}C^{+}$.

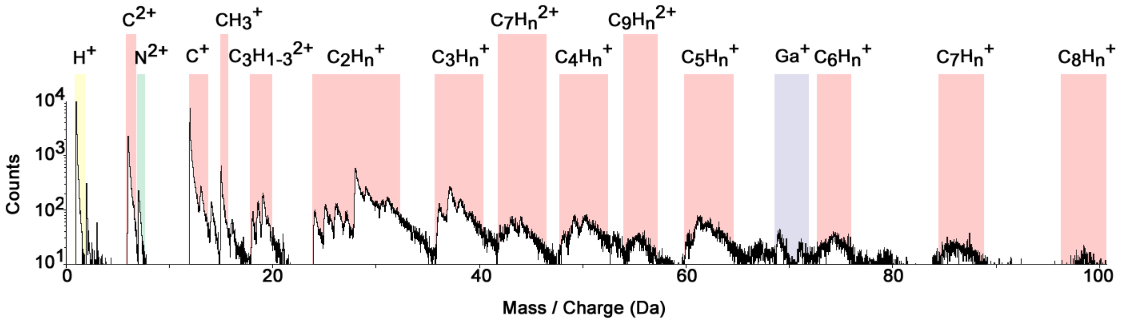

Figure S3. APT Mass Spectra of LR White Polymer Resin. APT mass spectrum from the embedding resin, with major ions labeled (n = 0, 1, 2 … 3-5). Note Ga^+ ions are present due to FIB preparation.

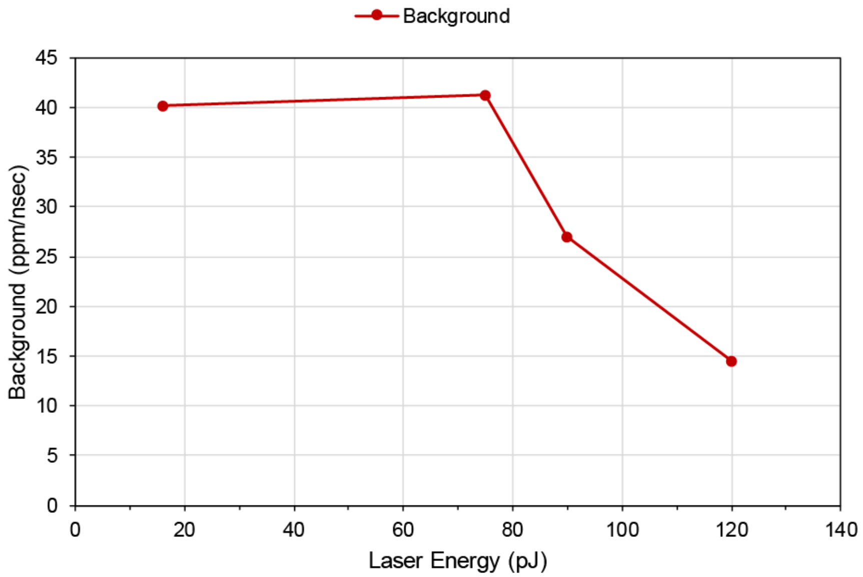

Figure S4. Effect of laser energy on background counts. The measured background signal is plotted for datasets obtained at 16, 75, 90 and 120 pJ. It is clear that below 90 pJ, the background level increases dramatically. This is the result of an increasing DC field needed to maintain evaporation at lower laser energies, and the resulting off-pulse DC evaporation it can produce.

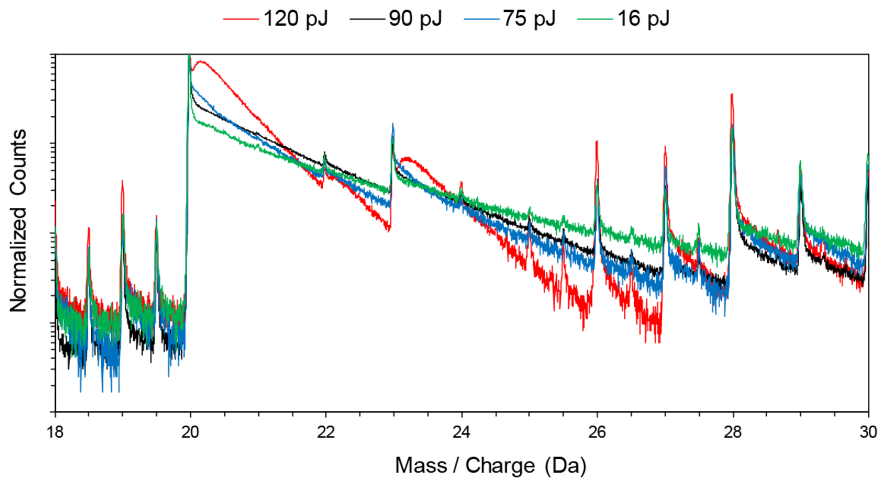

Figure S5. Effect of laser energy on the peaks in the mass spectrum. The mass spectrum showing the major $^{40}Ca^{2+}$ peak, normalized based the height of that peak, is plotted for datasets obtained at 16, 75, 90 and 120 pJ. The high laser energy of 120 pJ shows the lowest background levels, but also the largest thermal tails immediately following the peaks. The lowest laser energy of 16 pJ has the smallest tail in the vicinity of the peak, but it decays the least extending out to higher Da, due to the contribution from the high background level for that laser energy. Both the 75 pJ and 90 pJ datasets appear to provide the optimum balance between low background and short thermal tails.

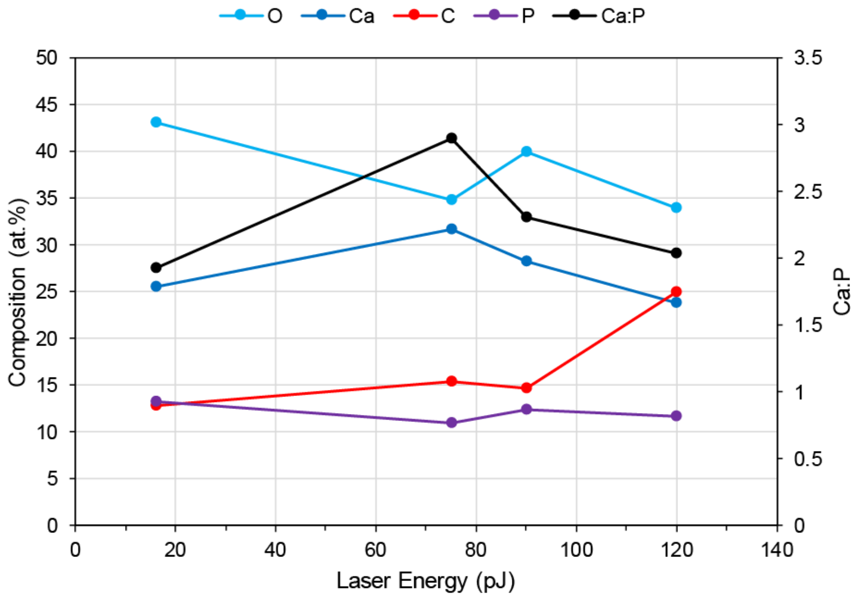

Figure S6. Effect of laser energy on measured composition. Compositions for major elements, as well as the Ca:P ratio, are plotted for datasets obtained at 16, 75, 90 and 120 pJ. No clear trends emerge over the range of 16-90 pJ, with only the 120 pJ data exhibiting a relatively high C concentration, and low O and Ca concentrations. This discrepancy may be due to sample-to-sample variability however.

Movie S1. APT of over 10 million ions of human bone showing Ca and C gradient maps, representing mineral and organic regions, respectively. (uploaded separately)
